# Impact of Prenatal Alcohol Exposure on the Development and Myocardium of Adult Mice: Morphometric Changes, Transcriptional Modulation of Genes Related to Cardiac Dysfunction, and Antioxidant Cardioprotection

**DOI:** 10.3390/antiox12020256

**Published:** 2023-01-23

**Authors:** Allan Luís Barboza Atum, Leonardo Paroche de Matos, Bruna Calixto de Jesus, Guilherme Rabelo Nasuk, Gabriel Almeida da Silva, Caio Perez Gomes, João Bosco Pesquero, Stella Regina Zamuner, José Antônio Silva Júnior

**Affiliations:** 1 Medicine Department, Universidade Nove de Julho—UNINOVE, Rua Vergueiro 249, São Paulo 01504-001, SP, Brazil; 2 Biophysics Department, Universidade Federal de São Paulo, Rua Pedro de Toledo 669, São Paulo 04039-032, SP, Brazil

**Keywords:** prenatal alcohol exposure, myocardium, signal transduction, oxidative stress, cardiac dysfunction

## Abstract

The impact of prenatal alcohol exposure (PAE) varies considerably between individuals, leading to morphological and genetic changes. However, minor changes usually go undetected in PAE children. We investigated PAE’s effects on gene transcription of genes related to cardiac dysfunction signaling in mouse myocardium and morphological changes. C57Bl/6 mice were subjected to a 10% PAE protocol. In postnatal days 2 and 60 (PN2 and PN60), morphometric measurements in the offspring were performed. Ventricular samples of the heart were collected in PN60 from male offspring for quantification of mRNA expression of 47 genes of nine myocardial signal transduction pathways related to cardiovascular dysfunction. Animals from the PAE group presented low birth weight than the Control group, but the differences were abolished in adult mice. In contrast, the mice’s size was similar in PN2; however, PAE mice were oversized at PN60 compared with the Control group. Cardiac and ventricular indexes were increased in PAE mice. PAE modulated the mRNA expression of 43 genes, especially increasing the expressions of genes essential for maladaptive tissue remodeling. PAE animals presented increased antioxidant enzyme activities in the myocardium. In summary, PAE animals presented morphometric changes, transcription of cardiac dysfunction-related genes, and increased antioxidant protection in the myocardium.

## 1. Introduction

Ethanol/alcohol consumption can induce several cardiac dysfunctions, such as cardiomyopathy and arrhythmias in adults. Increased consumption among women of childbearing age is a worrying health problem [1]. Furthermore, the legal responsibilities of mothers who drink alcohol during pregnancy have been established in many countries, and even the imputability of certain crimes to individuals with prenatal alcohol exposure (PAE) has been discussed [2,3]. The prevalence of alcohol use during pregnancy globally is 9.8% [4]. When ingested through pregnancy, alcohol freely crosses the placenta, reaching fetal organs even at low exposure (0.3–4.1 g/kg/week in humans), causing fetal organ damage [5]. In addition, prenatal alcohol exposure (PAE) increases cardiovascular risks in the fetus [6,7], leading to cardiac changes. These alterations can be seen directly in the myocardium when the structure and functionality of heart cells are compromised and indirectly related to the molecules and mechanisms that act in the functioning of the cardiovascular system [7,8].

Structural changes such as congenital heart diseases are observed in 38% of newborns with Fetal Alcohol Spectrum Disorders (FASD), a group of conditions identified in a person exposed to alcohol during embryonic development, especially related to neurodevelopmental and neurobehavior disorders [1,3]. In addition, congenital disabilities ranging from atrial or ventricular septal defects to aortic and valve malformations are observed in PAE individuals [9,10]. However, 80% of children born with FASD have no apparent physical symptoms, making it challenging to diagnose subtle heart disease [11]. Moreover, the absence of a biomarker used for FASD leads to misdiagnoses and has been a challenge for scientific and medical communities [12].

Antioxidant supplementation and early diagnosis are key to preventing congenital heart diseases induced by PAE [6]. Several bioactive molecules, such as resveratrol, folate, and ginger extract, are reported as potential mitigators of PAE effects in animals, and further studies must be conducted to determine the clinical applicability [6,13,14]. In addition to congenital heart diseases, the cardiovascular system’s functioning may be altered by PAE. Mechanistically, alcohol can interfere in excitation-contraction coupling, acting on the cytosolic calcium levels of cardiomyocytes and, therefore, their sensitivity to the cation [15]. Failure of this mechanism induces hypoxia and ischemia, resulting in heart defects or preterm delivery and miscarriages.

PAE can result in immediate and long-lasting toxic effects leading to impairments with lifetime repercussions. Adolescents with PAE are at risk for systemic inflammation [16]. A growing body of evidence has indicated that PAE elicits metabolic modifications [17] and cell death [18]. PAE may alter the redox state, consequently misbalancing ROS generation and causing an overall augmentation in oxidative stress [18].

Animal models to study PAE repercussions are powerful tools to analyze alcohol toxicity to organs, including the heart [19]. Alcohol causes a decrease in myocardial myofibrillar protein synthesis. Moreover, alcohol effects include reducing the number of myocytes and increasing the left ventricle size [20]. Furthermore, puppies of mice exposed to alcohol during pregnancy presented thinner ventricular walls and valvular defects [8] and high blood pressure resulting from increased renal and vascular function [21].

Therefore, to investigate the effect of PAE on the transcription of several cellular pathways and possible repercussions on the adult myocardium, we analyzed changes in morphometric measurements, mRNA expression of 47 cardiac dysfunctions-related genes of nine signal transduction pathways (angiogenesis, survival, apoptosis, kinetics of calcium, oxidative stress, hypertrophy, inflammation, extracellular matrix, and cellular metabolism), and activities of antioxidant enzymes in the myocardium of adult mice submitted to PAE.

## 2. Materials and Methods

### 2.1. Animals

Twenty isogenic mice (15 females and five males) of the C57Bl/ 6 strain, weighing 20–23 g, were used to generate the offspring. The animals were obtained at the Animal Facility of the Universidade Nove de Julho and kept confined in appropriate plastic boxes under a light cycle (light/dark cycle, 12 h/12 h). The temperature (21 ± 2 °C) and humidity were controlled, and food and water were ad libitum [20,21]. The Ethics Committee on the Use of Animals approved the study at the Universidade Nove de Julho (CEUA/UNINOVE) under CEUA number 9355120319 (ID 000115) on 19 March 2019.

**Figure 1:**
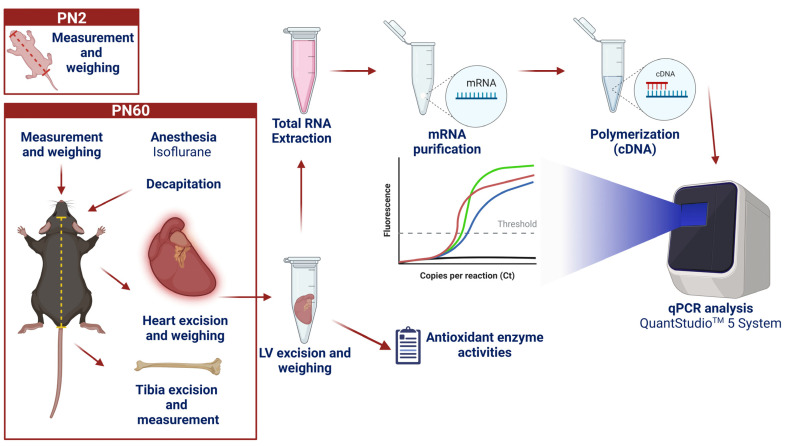
Representation of the sample collection, treatment, and analysis protocol in experimental groups. (Created with biorender^®^).

The females were randomized into two groups: the control group—CT (n = 3) and the prenatal alcohol exposure group—PAE (n = 12). The difference in sample size was due to the PAE group’s high death rate of newborn animals. Males were used only as breeders in a 1 male: 3 female ratio per cage. The mothers were euthanized after weaning. Experimental procedures are summarized in Figure 1.

### 2.2. PAE Protocol

**Figure 2:**
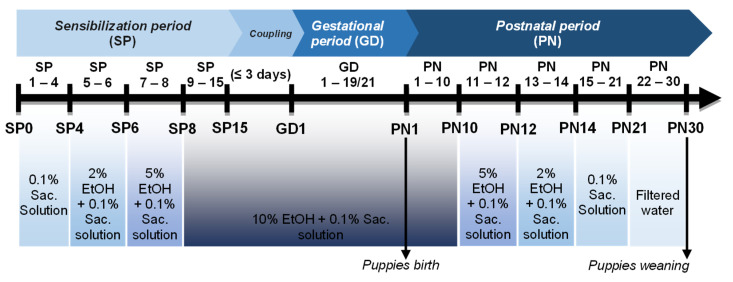
Representation of the prenatal alcohol exposure (PAE) protocol showing the timeline of alcohol consumption of breeders and the offspring until weaning.

The sensitization periods (SP1 to SP15) occurred as follows: In the first 4 days (SP1 to SP4), each female received a 0.1% saccharin (Sac) solution with changes every two days, avoiding fungal proliferation. Then, the alcoholic grade in the saccharin solution was gradually increased. On the following 2 days (SP5 and SP6), females received a 2% EtOH solution; on days SP7 and SP8, a 5% EtOH solution, and after that period, seven days (SP9 to SP15) of 10% EtOH solution and 0.1% Sac (Figure 2).

**Figure 3:**
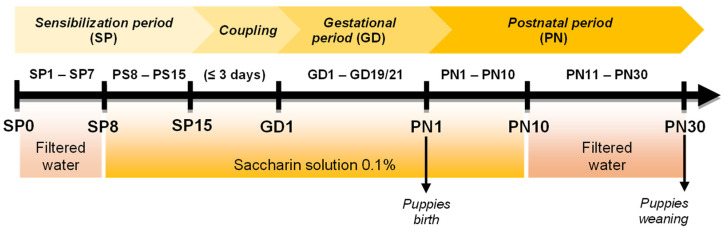
Representation of the Control protocol. The breeders and the offspring alternately received water and a sweetened solution with 0.1% saccharin.

After completing the sensitization protocol, the male was confined with the female for mating. During the copulation period, the couple shared the 10% EtOH alcoholic solution. The male was removed from the box after confirmation of pregnancy. From the gestational period (gestational day 1—GD1- to GD19/21) until the 10th day after the birth of the offspring (postnatal day 10—PN10), each female mother received the alcoholic solution in the concentration of 10% EtOH with an exchange carried out every two days. The 10% alcoholic solution elicited a maternal blood alcohol concentration of 80 mg/dL. As of PN11, the alcohol desensitization protocol began, so a 5% EtOH solution was made available to each female (Figure 2). On day PN13, there was a reduction to 2% EtOH solution, and from day PN15 to PN21, mice received saccharin solution. Finally, the females received only filtered water from day PN22 until weaning (PN30) [22,23]. Thus, control groups received the same proportion of water and saccharin at each period established to PAE but without alcohol sensibilization and administration (Figure 3).

### 2.3. Biological Samples and Morphometric Measurements

For size calculations, ponderal indexes were determined using body weight, snout-rump length, and tibia length measurements. Ventricular and cardiac indexes were calculated by the coefficient between tissue weight and tibial length (heart/tibia and left ventricle/tibia).

First, in PN2, male mice were anesthetized with isoflurane inhalation, and then morphological measurements were performed. After the measurements, the animals were placed with their respective mothers and siblings. In PN60, mice were anesthetized with isoflurane inhalation and euthanized after trichotomy. A left thoracotomy was performed, and the heart was exteriorized. The hearts and left ventricle (LV) samples were collected and immediately weighed. Then, LV samples were washed and prepared for measurements of antioxidant activities or snap-frozen for qPCR analyses. Next, the left tibia of each animal was exposed, and its size was measured with a caliper, taking the medial malleolus and medial condyle as a reference. Then, ratios were calculated between heart and left ventricle weights in milligrams and the length of the left tibia in millimeters [24].

As the number of animals born per gestation was unpredictable, if there were surplus animals, they were also euthanized, and the organs were collected for further analyses unrelated to this study.

### 2.4. Quantitative PCR

The mRNA expression of 42 genes, previously selected [25], was analyzed according to their participation in the signal transduction pathway related to several cardiac pathways, using a customized TaqMan^®^ plate (Custom TaqMan^®^ Plates—Life Technologies, Carlsbad, CA, USA). Primer sequences used for mRNA quantification are shown in Table S1-Supplementary Material.

#### 2.4.1. RNA Extraction

Left ventricular (LV) samples (n = 5 per group) from both groups were homogenized in 1 mL of Trizol^®^ Reagent (Life Technologies, Carlsbad, CA, USA) and incubated for 5 min at room temperature for complete dissociation of the nucleoprotein complexes. Then, 200 µL of refrigerated chloroform was added to the homogenates, vortexed for 15 s, and incubated at room temperature for 10 min. After incubation, the samples were centrifuged for 15 min (12,000× *g* at 4 °C).

The resulting aqueous phase of each sample was removed and transferred to sterile 1.5 mL Eppendorf tubes. A 500 µL of refrigerated isopropanol was added for RNA precipitation, and the sampler was kept at room temperature for 10 min and then centrifuged (12,000× *g*/4 °C/10 min). The supernatant was discarded, and the RNA pellets were washed with 1 mL of refrigerated 75% ethanol (prepared with water treated with diethylpyrocarbonate, DEPC, 0.01%). Again, the samples were centrifuged (7500× *g*/4 °C/5 min), and the supernatant was discarded. Finally, the pellets were air-dried and resuspended with 15 µL of DNase I/RNase-free water (Invitrogen, Carlsbad, CA, USA).

Total RNA quantifications were obtained using the NanoDrop ND-2000 spectrophotometer (NanoDrop Products, Wilmington, DE, USA), where 1U at A260 corresponds to 40 µg of RNA/mL. Only samples free of contaminants (A260/A230 ~1.8) and proteins (A260/A280 = 1.8—2.0) were used. One µg of total RNA was incubated with 1 unit of DNase I/RNase free and a solution of 20 mM Tris-HCl, pH 8.4, and 2 mM MgCl2 for 15 min at 37 °C to eliminate genomic DNA contamination, followed by incubation at 65 °C for 10 min for DNase I inactivation. The samples were kept at −80 °C to further cDNA synthesis.

#### 2.4.2. Reverse Transcription

For reverse transcription, the SuperScript^®^ IV Reverse Transcriptase Kit (Invitrogen, Carlsbad, CA, USA) was used. Initially, 1 µg of RNA was transferred to microtubes, then a volume of 3 µL of a solution containing 1 µL of 50 µM Oligo d(T)20 Primer, 1 µL 10 mM dNTP mix, and 1 µL of DNase I/RNase Free (Invitrogen, Carlsbad, CA, USA) was added in each tube. Then, the samples were incubated in a thermocycler for 5 min at 65 °C, incubated on ice for 1 min, and added 7 µL of a solution containing 1 µL of Ribonuclease Inhibitor, 1 µL of SuperScript^®^ IV Reverse Transcriptase (200 U/ µL), 1 µL 100 mM DTT and 4 µL 5× SSIV Buffer (Invitrogen, Carlsbad, CA, USA). The resulting solution was subjected to two incubation cycles: 10 min at 55 °C and 10 min at 80 °C. Next, 1 µL of RNase H (Invitrogen, Carlsbad, CA, USA) was added and incubated for 20 min at 37 °C to remove residual RNA. After the reaction, the cDNA samples were kept at −20 °C.

#### 2.4.3. Quantitative Real-Time Polymerization Chain Reaction (PCR)—qPCR

Amplification and data acquisition were performed using TaqMan customized plates (ID 1409183) in a Quantstudio™ 5 System equipment (Applied Biosystems, Carlsbad, CA, USA). The samples were applied in duplicate and incubated at 95 °C for 20 s and passed through 40 thermal cycles at 95 °C for 3 s and 60 °C for 30 s. The experiments were repeated 3 times. The reactions were submitted to the same conditions and normalized by the passive reference dye ROX signal to correct fluctuations in the reading due to volume variations and evaporation throughout the reaction. The result, expressed in CT value, referred to the number of PCR cycles required for the fluorescent signal to reach the detection threshold. In addition, the differentially expressed genes were normalized by the expression of the housekeeping gene *18S subunit of the ribosomal RNA*, whose expression was unaltered under the experimental conditions. The QuantStudio™ Design & Analysis software version 1.3.1 (Applied Biosystems, Carlsbad, CA, USA) was used for data processing. The ΔCT values were determined by subtracting the mean CT value of the target gene mRNA from the mean CT value of the *18S rRNA* housekeeping gene. The 2^−ΔΔCT^ parameter was used to represent the relative expression data [23].

### 2.5. Antioxidant Enzymes Activities

Left ventricle homogenates were used to quantify superoxide dismutase (SOD), catalase (CAT), and glutathione peroxidase (GPx) activities in the experimental groups in PN60 (n = 5 LV/group). SOD activity was analyzed using the pyrogallol autoxidation assay. CAT activity was determined by adding 0.03 M H_2_O_2_ to the homogenate, and the absorption was analyzed at 240 nm. Finally, the activity of GPx was determined by a GSH reduction coupled with NADPH oxidation by glutathione reductase. NADPH oxidation was monitored at 339 nm.

### 2.6. Statistical Analysis

Statistical calculations were performed using the IBM Corp. application. IBM SPSS Statistics for Windows, Version 25.0 (Armonk, NY: IBM Corp., 2017). To verify normality and error variances, the Shapiro-Wilk test was used. The measurements from females (average consumption, gestational weight gain, and the number of pups) and offspring (weight, length, heart weight, left ventricle weight, and heart/tibia—LV/tibia) were presented when parametric, by mean ± standard error of the mean (SEM) and, when not, by median and interquartile ranges 25–75%. For comparison between groups, Student’s *t*-test and Mann-Whitney test were performed, with a significance of ≤0.05. The survival analysis was obtained using the Mantel-Cox test. Finally, the Student’s *t*-test was performed to compare gene expression and, when necessary, supplemented with the Welch correction test. A *p*-value ≤ 0.05 was considered significant, and results were expressed as mean ± standard error of the mean (SEM).

## 3. Results

**Figure 4:**
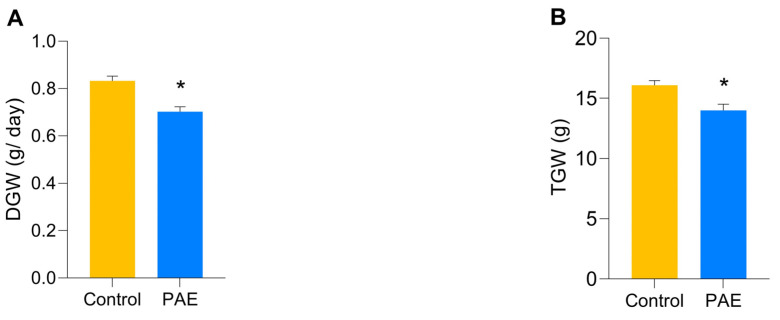
Total and daily gestational weight gain in the sample groups, n= 5 per group. (**A**) Daily gestational weight gain (DWG) in grams per day (g/day); (**B**) Total gestational weight gain (TWG) in grams (g); A significant reduction was observed in the PAE group in relation to the Control group in both analyzed criteria. (MED ± SEM). * *p* ≤ 0.05 versus Control group, determined by *t*-test for independent samples.

The average daily drinking of the female PAE progenitors (9.74 ± 0.33 mL/day) and the Control group (10.63 ± 0.13 mL/day) was similar (*p* = 0.121; Table 1). Differences in pre-gestational weight gain (PGW) between PAE females and the Control group were not observed (*p* = 0.086). Two measurements were made regarding gestational weight gain: the daily (DGW) and the total (TGW) gestational weight. Findings showed that PAE females presented significantly lower DGW (0.70 ± 0.02 g/day; *p* = 0.0005) than the Control group (0.83 ± 0.02 g/day; Figure 4A). These data were totalized and accounted for the TGW to significantly diminished PAE values (14.0 ± 0.5 g; *p* = 0.012) compared to the Control group (16.1 ± 0.4; Figure 4B).

**Figure 5:**
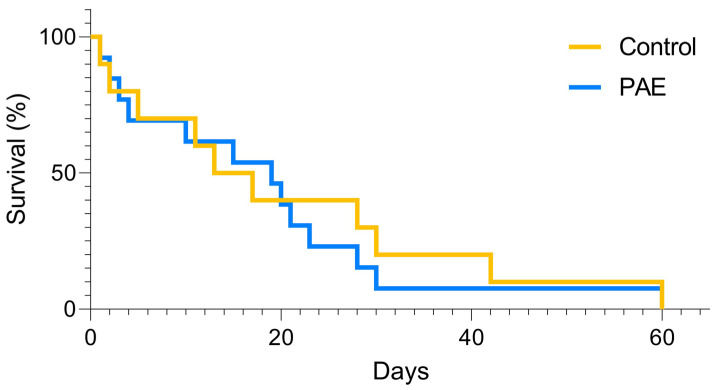
Mortality/survival curve between groups, n = 50 animals per group. There is no statistically significant difference between PAE and control groups. Obtained through the Mantel-Cox test.

**Figure 6:**
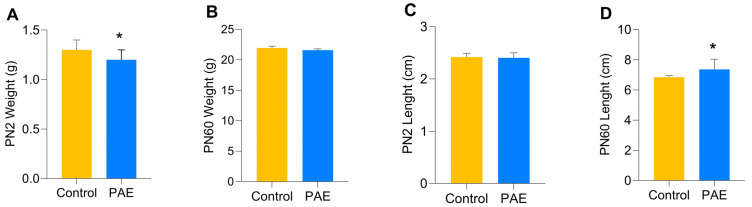
Morphometric data of the pups in the experimental groups, n = 5 per group. (**A**) Body weight at birth (PN2); (**B**) body weight (PN60); (**C**) body length at birth (PN2); (**D**) body length (PN60). Values in grams (g) and centimeters (cm) (M_d_, IQR 25%–75%). * *p* ≤ 0.05 versus Control group, determined by Mann-Whitney Test (U).

After delivery, the number of offspring and deaths per litter in the PAE group were comparable to the Control group (Table 1). In addition, the offspring’s survival curve presented no differences between the experimental groups after the Mantel-Cox test (*p* = 0.1723; Figure 5). Fifty male mice per group were used for morphometric measurements. The weighing was performed at two stages, PN2 and PN60. Comparison of post-birth weight at PN2 revealed that PAE mice presented significantly decreased weight than the Control group [1.2 g (1.0–1.3) vs. 1.3 g (1.2–1.4); *p* = 0.0037, respectively; Figure 6A]. However, comparing animals at PN60, no differences in body weight were observed between experimental groups [21.45 g (20.68–22.50) PAE vs. 22.15 g (21.33–22.98)—Control group: Figure 6B].

The snout-rump length was used to evaluate the animal’s size. PN2 presented no differences between the experimental groups [2.41 cm (2.24–2.50) PAE vs. 2.42 cm (2.34–2.49) in the Control group (*p* = 0.6241) Figure 6C]. However, in PN60, interestingly, the PAE animals presented increased size [7.36 cm (6.71–8.04), *p* = 0.0015] compared to the Control group [6.85 cm (6.71–6.95)] (Figure 6D).

**Figure 7:**
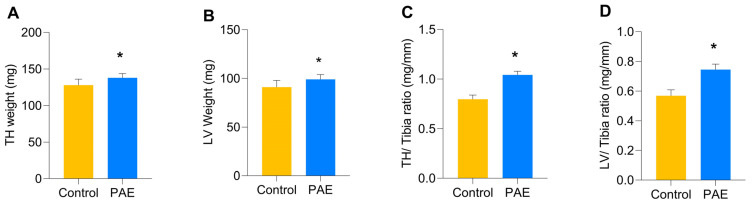
Morphometric data of the total heart (TH) and left ventricle (LV) in the sample groups. (**A**) TH weight; (**B**) LV weight; (**C**) TH/Tibia ratio; (**D**) LV/Tibia Ratio. There is a statistically significant increase in all parameters observed in the PAE group compared to the Control group. (Md, IQR 25–75%). Values in milligrams (mg) and millimeters (mm). n = 5 per group. * *p* ≤ 0.05 versus Control group, determined by Mann-Whitney Test (U).

Animals from the PAE group showed increased heart and left ventricle weights (*p* < 0.0001) compared to the Control group. The heart weight mean was 139.5 mg (136–148) in the PAE and 134 mg (128–137) in the Control group (Figure 7A). Similarly, the LV absolute weight in the PAE group was 99.5 mg (96–108), and in the Control group, the weight was 96 mg (90–99) (Figure 7B).

The ratio between heart or ventricular weight and the tibial length was used to determine cardiac indexes at PN60. Increased indexes were found in PAE mice compared to the Control. PAE mice presented a heart index of 1.05 mg/mm (1.02–1.11) compared to the Control group [0.77 mg/mm (0.76–0.78); Figure 7C]. Likewise, the ventricular index was increased in PAE mice [0.75 mg/mm (0.72–0, 81)] compared to the Control group [0.54 mg/mm (0.53–0.55; Figure 7D)].

The gene expression results in the experimental groups at PN60 were given by the relative transcript levels normalized to the *18S rRNA* gene. Genes that presented increased (upregulated) or decreased (downregulated) expression compared to the Control group are shown in Table 2. Data showed an accentuated modulation of most genes analyzed. Thus, the PAE induced the mRNA upregulation of 32 genes (68.1%), whereas eleven genes presented downregulation (23,4%), and four genes remained unaltered (8.5%) on the 60th-day post-birth.

A decrease of 0.6-fold in the mRNA expression of *Vascular endothelial growth factor A* (*VEGFA*) and an increase of 2.1-fold in the expression of cell survival *AKT serine/threonine kinase-1* genes were observed in PAE animals compared to the Control group. The cell apoptosis signal transduction pathway was evaluated by the quantification of mRNA expression of the genes *BCL2-associated Protein X (Bax*), *death receptor cell surface Fas (Fas), mitogen-activated Protein Kinases 1* and *14 (Mapk1)* and *(Mapk14),* and *tumor -protein P53 (Tp53)*. The PAE group presented a significant upregulation in mRNA expression of all apoptosis-related genes, except for the *Fas* gene, which remained statistically unaltered. 

The gene coding for *mitogen-activated protein Kinase 14* showed an increase of 4.1-fold in mRNA expression compared to the Control group. The *Tp53, Mapk1,* and *Bax* genes also presented significant mRNA expression of 0.8-, 1.2-, and 1.7-fold, respectively, compared with the Control group.

Regarding calcium kinetics, in the PAE group, a significant reduction of 0.6-fold in both expressions of *Calsequestrin 2 (Casq2)* and *Solute Exchanger Family (Na-Ca) 8, member A1 (Slc8a1)* mRNAs was observed compared to the Control group. In turn, the PAE group showed considerable increases in mRNA expression of 2.6-, 2.4-, and 2.0-fold in *ATPase, Ca2+ transport, cardiac muscle, slow contraction 2 (Atp2a2), Receptor 2 of ryanodine, (RyR 2)* and *phospholamban (Pln),* respectively, compared to the Control group.

Alcohol, especially in the PAE, is an important event increasing oxidative stress in the body. The mRNA expression of *members 1A and 1B of the family of heat shock proteins—Hsp70 (Hspa1a/1b)* and of antioxidants enzymes *catalase (Cat), Superoxide Dismutase 1 (Sod1),* and *Glutathione Peroxidase 4 (Gpx4)* were accessed. A significant increase was observed in all genes related to oxidative stress in the PAE group. The mRNA expression of the *Hspa1a/1b* gene showed an increase of 1.9- fold compared to the Control. The genes *Gpx4, Sod1,* and *Cat* present increased mRNA expression of 1.5-, 1.4-, and 1.1-fold, respectively, compared to the Control group.

A significant reduction in the mRNA expression of glycolysis-related genes was observed. The *Glyceraldehyde-3-Phosphate Dehydrogenase* (*Gapdh*; 0.6-fold), *Hexokinase 1* (*Hk1;* 0.7-fold), *and muscle Phosphofructokinase* (*Pfkm*; 0.4-fold) mRNA expressions were also decreased when compared to the Control group. On the other hand, a significant increase in the mRNA expression of *NAD+Ubiquinone Oxidoreductase Subunit A3* (*Ndufa3;* 1.2-fold), *Glucose transporter type 1* (*Slc2a1;* 2.0-fold), *Tafazzin (Taz;* 1.3-fold), and *Uncoupling Protein 2* (*Ucp-2*; 1.5-fold) genes were observed compared to the Control group.

The inflammation pathway was also assessed, and a significantly increased mRNA expression in all genes analyzed in the PAE group was observed. In addition, increased mRNA expressions of *IL-6* (2.0-fold), *Tnfrsf1a* (1.7-fold), and *TNF* (1.2-fold) were found compared to the Control group.

Moreover, the PAE group presented downregulation of both mRNA expression of the *protein 2 similar to Calcineurin B* (*Chp2*)*, Angiotensin II receptor, type 1a* (*Agtr1a*)*,* and *α-myosin heavy chain* (*Myh6*) genes by 0.8-, 0.8-, and 0.7-fold compared to the Control group. In contrast, the genes of the *β-myosin heavy chain* (*Myh7*) and *protein 1 binding to Calcineurin* (*Cabin1*) in the PAE group presented increments of 1.3-fold and 1.7-fold in mRNA expression, respectively, compared to the Control group. In addition, the mRNA expressions of *activated T cell nuclear factor, cytoplasmic, dependent on Calcineurin 3* (*Nfatc3*)*, insulin-like growth factor 1* (*Igf1*)*,* and *protein Kinase, Kinase, Kinase, 2 activated by Mitogen* (*Map3k2*) were increased by 1.6-, 1.2-, and 0.8-fold, respectively, compared to the Control group.

The mRNA expressions of genes encoding natriuretic peptides and protein kinases C isoforms, important cardiac hypertrophy markers, were significantly increased in the PAE group compared to the Control group. Notably, the *natriuretic peptide B* (*Nppb*) gene presented the second-highest increase in relative mRNA expression (3.2-fold) compared to the Control group. Furthermore, in comparison to the Control group, the genes of the *natriuretic peptide A (Nppa), protein Kinase C beta (Prkcb), alpha (Prkca),* and *gamma (Prkcg)* also presented elevated mRNA expression of 2.6-, 3.1-, 2.8- and 1.3-fold, respectively, in PAE group.

Accordingly, like the genes related to cardiac hypertrophy, extracellular matrix genes showed significant changes in mRNA expression due to PAE. Therefore, the mRNA expressions of *Col1a1, Col3a1, Tgfb1,* and *Mmp9* genes were evaluated to assess the PAE effect on cardiac extracellular matrix dynamics. In the PAE group, the *type III collagen α 1* mRNA expression (*Col3a1*) was unaltered compared to the Control group. Oppositely, the PAE group presented an increment of 2.8-fold in the *transforming growth factor β 1* (*Tgfb1*) mRNA expression. In addition, significant increases of 2.8-fold in the mRNA expression of the *matrix metalloproteinase 9* (*Mmp9*) gene and 1.7-fold in the *alpha 1 type I collagen* (*Col1a1*) mRNA expression were observed in comparison to the Control group.

In agreement with the transcriptional changes of oxidative stress-related genes, augmented activities of antioxidant enzymes SOD, CAT, and GPx were found in PAE myocardium in PN60 (Table 3).

## 4. Discussion

The toxic effects of alcohol on embryo development are widely reported. Alcohol readily crosses the placental and blood-brain barrier and impairs cell development in several organs [26]. Although PAE induces the phenotypes observed in FASD through multiple mechanisms and at different developmental stages, the fetal damage extension depends on the dose threshold and duration of exposure [27,28]. Some induced alterations are regularly observed after birth. However, silent ones continue to occur, as suggested by the results of this study, and may lead to long-lasting effects in adulthood. Our findings suggested that PAE induced developmental changes and transcriptional modulation in the myocardium.

Moreover, the numerous molecular changes were similar to those observed in experimentally infarcted animals [29]. In both conditions, transcription activation of dysfunction-related genes and possible translation of these transcripts lead to cell and tissue damage. Thus, our study highlights the silent harmful effect of PAE on the adult mice heart.

Alcohol consumption did not affect pre-pregnancy weight [30], as pointed out by our data. However, daily and total weight losses were observed in PAE dams throughout gestation. These observations are worthy of consideration as studies suggest that decreased gestational weight during PAE is associated with the development of coronary heart disease, hypertension, type II diabetes, and asthma in the offspring [31,32]. Moreover, [33], in a cohort study of the association between PAE and low birth weight, it was found that the fetus of women who drank alcohol in the first trimester of pregnancy was most sensitive to weight loss caused by PAE, even at levels considered negligible (less than two units/week).

Several studies reported that PAE was sufficient at low to moderate doses to result in animal intrauterine growth restriction (IUGR) [19,34]. Incidentally, PAE induces IUGR in humans [32,33,34,35]. Our protocol showed that alcohol, administered continuously throughout gestation and 10 days after delivery, resulted in PAE group offspring with low weight after birth. However, in PN60, the PAE mice presented a similar weight to the Control group. Several studies have shown that weight gain in early adulthood is related to the accelerated growth of recovery at the onset of puberty [21,36]. Unlike the weight values, the animals’ size differed from the experimental groups at PN60, whereas PAE animals were larger than the Control animals. Our protocol did not observe differences in offspring mortality rates between the experimental groups. This observation corroborates studies that show that PAE does not alter the mortality rates of puppies exposed to low to moderate doses of alcohol [23].

Cardiac hypertrophy after PAE has been reported, although controversial, in several studies in animals and humans. For example, using alcohol administration at dose of 6.36% *v/v*, [37] found that 120-day-old Sprague-Dawley rats exhibited left ventricular hypertrophy and fibrosis but not PN30. In opposition, [17] found increased cardiac hypertrophy in male animals at PN30. Our results indicated that, in PN60, cardiac and left ventricular indexes were higher in animals in the PAE group than in the Control group, suggesting mild cardiac hypertrophy. These data, in PN60, corroborated studies indicating that PAE altered the cardiac structure and led to a hypertrophic state, even in early rodents’ adulthood [37] and in humans [38]. Notably, the correlation between PAE and cardiac hypertrophy is clinically relevant because left ventricular hypertrophy is the strongest predictor of adverse cardiovascular events, regardless of age, sex, and blood pressure [17,36,39]. Cardiovascular diseases and associated renal damage occur most frequently in late adulthood and early senescence. Then, modifications induced by prenatal alcohol exposure may trigger continuous, silent processes that could predispose these animals to cardiovascular changes at an older age [17,37,40].

The natriuretic peptides, gold standards biomarkers in heart failure (HF) [41], are also characterized as hallmarks of cardiac hypertrophy [42,43]. Our protocol analyzed the mRNA expression of genes encoding *ANP* (*Nppa*) *and BNP* (*Nppb*). These genes are mainly transcribed in the myocytes of the atria and ventricles, respectively [44]. However, due to stress stimuli, fetal reprogramming of these peptides avows their ventricular re-expression in response to stretching the myocardium [41,45], which often occurs in heart failure (HF). Interestingly, our results indicated that a robust mRNA expression of natriuretic factors, especially *BNP*, was observed in the PAE ventricular samples. To our best knowledge, modulation of natriuretic peptide expression has not been observed with PAE protocols. In addition to natriuresis, diuresis, and vasodilation, BNP acts directly on the heart [46], providing compensatory protection by inhibiting myocardial cell death as an attempted effort to reduce cardiac hypertrophy [47].

In our study, endothelin-1 (Edn1) did not present altered expression levels. Indeed, [48] did not detect significant changes in the expression and serum concentration of endothelin 1 in the carotid artery of adult Wistar rats exposed to an alcohol concentration of 20% (*v/v*), even after 10 weeks. Furthermore, using lower alcohol concentration in our protocol, the *Edn1* mRNA expression remained unchanged after PAEThe renin-angiotensin-aldosterone system (RAAS) contribution to cardiac hypertrophy is widely evidenced. A study by [49] showed that in vitro and in vivo, alcohol-induced cardiac cell death could be triggered by nitrative stress from a PKCβ1- and NOX-dependent pathway activated by Ang II. AT1R blockade prevented nitrative cardiac damage, cell death, and remodeling. We suggest that upregulation of mRNA expression of *angiotensin-converting enzyme* (*ACE*) and *Agtr1a* (*AT1R)* genes may increase Ang II availability and effectivity, increasing heart stress.

*Alpha-myosin heavy chain* (*Myh6*) mRNA expression was negatively regulated in PAE mice. Hypertrophy is associated with an induction of β-MHC at the expense of α-MHC; therefore, this phenotype change is evidence of cardiac hypertrophy. Furthermore, a decreased *Myh6* expression and the re-expression of *beta-myosin heavy chain (Myh7*) mRNAs directly impair myocardial contractile capacity after MI [50,51].

Studies on PAE attribute a cardioprotective role to IGF-1. For example, Chen and colleagues [52], using fetal cardiomyocytes isolated from rats treated with 0.2% alcohol, observed that an increase in *IGF-1* expression was related to a decrease in apoptotic cell death. A study by [53] reported that *IGF-1* mRNA upregulation caused accelerated maturation and consequent enlargement of fetal cardiomyocytes isolated from sheep exposed to alcohol during gestation and prevented apoptosis. Moreover, an increased mRNA expression of *IGF-1* has already been described in other tissues undergoing apoptosis [54,55]. In fact, the *IGF-1* mRNA expression augmentation observed in our study could be a compensatory response of the myocardium to alcohol exposure. Indeed, some studies have observed that increased *IGF-1* expression after PAE is directly related to *protein C kinase (PKC*) expression [56]. Other authors have shown that PAE at 5% *v/v* significantly increase neuronal *PKC-alpha* and *PKC-gamma* gene expression [57]. Moreover, protein C kinases are indirectly activated by alcohol after PAE [58]. These shreds of evidence suggest that the increase in expression of *Prkca* and *Prkcb* genes, together with augmented *mitogen-activated protein kinases (MAPKs*) mRNA expressions observed in our study, may play a critical role in transmitting and regulating intracellular signals. The MAPK signaling pathway includes the activation of kinases and their amplification actions [59], therefore transducing the signal for reprogramming and/or modulating cardiac gene expression [60,61].

The increase in cardiac *matrix metalloproteinase-9 (Mmp-9)* expression is associated with collagen I and III maturation in individuals with HF, indicating the importance of these enzymes in the activation and deposition of collagen in the fibrosis process [62]. The increase in *Mmp9* gene expression, along with an increased *TGF-β1* mRNA expression—a persistent stimulus in the repair phase of hypertrophy and an important pro-fibrotic cytokine—reinforce the hypertrophic environment triggered by PAE. For instance, anti-TGF-β1 therapy attenuates cardiac remodeling [62]. The increased expression of the mRNAs of *collagen I, TGF-β1,* and *matrix metalloproteinase 9* in PAE animals indicates a signal transduction mechanism that might suggest the cardiac repair process [63]. Indeed, the observed upregulation of calcium kinetic- and metabolism-related genes also evidenced the damage extension of PAE in the myocardium of adult mice.

Finally, the mRNA expression of antioxidant enzymes increased in PAE mice. A study performed by [64] found diminished mRNA expression of SOD, CAT, and GPX4 in PAE animals. However, the authors used samples harvested at a different endpoint (GD18) and a different PAE protocol. Shirpoor et al. [65] reported that cardiac *HSP70* expression increased in PAE animals at PN21 and PN90. The authors observed, together with other data, that there was activation of a protective pathway in a tentative to reduce damage related to PAE. Accordingly, we might assume that increased gene expression and activities of antioxidant enzymes, along with increased *HSP70* mRNA expression, may alleviate oxidative stress in the myocardium.

An interesting and comprehensive review [66] reported genetic and environmental influences on alcohol consumption. Common genetic changes in humans related to alcohol metabolism are polymorphisms of alcohol and aldehyde dehydrogenases (ADH and ALDH, respectively). Drugs and gene therapy have been tested to reduce enzyme activities and alcohol intake. An environmental influence raised by the authors is precocious exposure to alcohol, especially since PAE predisposes to alcohol abuse and dependence later in life [66]. Early exposure to alcohol can alter the brain development pattern, interfering with several tasks’ performance in adults [66], besides experiencing lifelong disabilities. There are still some limitations in the present study. First, we did not address protein expression. Due to the high number of genes analyzed, our laboratory is doing antibody screening to access relevant expression differences to carry out protein experiments. A submitted work [67] addressed molecular changes in peptidergic systems in the hippocampus and myocardium after PAE. As a cross-sectional study, and despite the morphometric measurements performed in PN2, the complete protocol was performed with only one endpoint (PN60). We hope to show new results soon.

## 5. Conclusions

This study showed that PAE promoted morphometric changes, fetal gene reprogramming, altered mRNA expression of several genes related to cardiac dysfunction, and increased antioxidant enzyme activities in the myocardium from male mice in PN60. Two significant conclusions are possible to achieve based on our data. First, the evidence of a reprogrammed mRNA expression profile of genes encoding fetal contractile proteins and gold standards biomarkers for cardiac hypertrophy and increased cardiac indexes suggests a hypertrophic state of adult PAE animals. Then, the transcriptional activation of physiopathological biomarkers of different signal transduction pathways, together with the increased antioxidant activities, evidence the stressor role of alcohol when administered as PAE in the heart of adult mice. In addition, the dysfunctional profile of gene expression observed in the myocardium, combined with unhealthy habits commonly observed in adulthood, such as excessive alcohol consumption, smoking, abusive use of other drugs, and ingestion of ultra-processed foods, might facilitate the establishment and progression of cardiovascular diseases, worsening the prognosis.

## Figures and Tables

**Table 1:** Pregnancy and litter data of C57Bl6 mice submitted to 10% PAE protocol.

Maternal Data	Control	PAE	*p* Value
Daily consumption (mL/day)	10.63 ± 0.13	9.74 ± 0.33	0.121
Number of litters	10	16	
Number of puppies	78	113	
Average litter size	7.8 ± 0.25	7.1 ± 0.46	0.245
Total death	24	61	
Death per litter	2.4 ± 0.64	3.8 ± 0.80	0.223

Data are shown as mean ± SEM of 50 animals per group.

**Table 2:** Relative expression mRNA; PAE group compared to the Control group.

Gene	Control	PAE	*p* Value
*Akt1*	1.0	3.1 ± 0.12 *	<0.001
*Vegfa*	1.0	0.4 ± 0.02 *	<0.001
*Bax*	1.0	2.7 ± 0.05 *	<0.001
*Fas*	1.0	1.1 ± 0.05	0.1170
*Mapk1*	1.0	2.2 ± 0.05 *	<0.001
*Mapk14*	1.0	5.1 ± 0.05 *	<0.001
*Tp53*	1.0	1.8 ± 0.03 *	<0.001
*Atp2a2*	1.0	3.6 ± 0.07 *	<0.001
*Casq2*	1.0	0.4 ± 0.02 *	<0.001
*Pln*	1.0	3.0 ± 0.15 *	<0.001
*RyR-2*	1.0	3.4 ± 0.16 *	<0.001
*Slc8a1*	1.0	0.4 ± 0.04 *	<0.001
*Cat*	1.0	2.1 ± 0.18 *	<0.001
*Gpx4*	1.0	2.5 ± 0.13 *	<0.001
*Hspa1a/b*	1.0	2.9 ± 0.24 *	<0.001
*Sod1*	1.0	2.4 ± 0.05 *	<0.001
*Ace*	1.0	0.5 ± 0.01 *	<0.001
*Ace2*	1.0	0.2 ± 0.02 *	<0.001
*Agtr1a*	1.0	0.2 ± 0.02 *	<0.001
*Cabin1*	1.0	2.7 ± 0.17 *	<0.001
*Chp2*	1.0	0.2 ± 0.05 *	<0.001
*Edn1*	1.0	1.0 ± 0.12	0.511
*Igf1*	1.0	2.2 ± 0.07 *	<0.001
*Map3k2*	1.0	1.8 ± 0.04 *	<0.001
*Myh6*	1.0	0.3 ± 0.01 *	<0.001
*Myh7*	1.0	2.3 ± 0.13 *	<0.001
*Nfatc3*	1.0	2.6 ± 0.05 *	<0.001
*Nppa*	1.0	3.6 ± 0.28 *	<0.001
*Nppb*	1.0	4.2 ± 0.16 *	<0.001
*Prkca*	1.0	2.8 ± 0.19 *	<0.001
*Prkcb*	1.0	3.1 ± 0.23 *	<0.001
*Prkcg*	1.0	2.3 ± 0.09 *	<0.001
*IL-6*	1.0	3.0 ± 0.15 *	<0.001
*Tnfrsf1a*	1.0	2.7 ± 0.12 *	<0.001
*Tnf*	1.0	2.2 ± 0.10 *	<0.001
*Col1a1*	1.0	1.7 ± 0.11 *	<0.001
*Col3a1*	1.0	1.1 ± 0.07	0.3694
*Mmp9*	1.0	3.8 ± 0.21 *	<0.001
*Tgfb1*	1.0	3.8 ± 0.22 *	<0.001
*Tnc*	1.0	1.0 ± 0.03	0.3026
*Gapdh*	1.0	0.4 ± 0.06 *	<0.001
*Hk1*	1.0	0.3 ± 0.02 *	<0.001
*Ndufa3*	1.0	2.2 ± 0.09 *	<0.001
*Pfkm*	1.0	0.6 ± 0.03 *	<0.001
*Slc2a1*	1.0	3.0 ± 0.11 *	<0.001
*Taz*	1.0	2.3 ± 0.07 *	<0.001
*Ucp-2*	1.0	2.5 ± 0.04 *	<0.001

Data are shown as mean ± SEM of *n = 5* samples *per* experimental group. * *p* ≤ 0.05 versus Control group. The *p* values were determined by *t*-test for independent samples with Welch correction, when necessary.

**Table 3:** Effect of PAE on the antioxidant enzymes activities in LV of C57Bl/6 mice.

	Control	PAE
SOD (U/mg protein)	142.11 ± 27.45	199.20 ± 20.91 *
CAT (mol H2O2/min mg protein)	23.50 ± 5.30	33.9 ± 3.14 *
GPx (nmol NADPH/mg protein/min)	50.40 ± 19.26	89.6± 12.8 *

Data are shown as mean ± SEM of 5 animals per group. ^*^ *p* ≤ 0.05 vs. Control male.

## Data Availability

Data are contained within the article and Supplementary Material.

## Supplementary Materials

The following supporting information can be downloaded at: https://www.mdpi.com/article/10.3390/antiox12020256/s1, Table S1: Selected genes for mRNA quantification (Custom TaqMan^®^ Plates).
