# Supplementary material for: Impact of Prenatal Alcohol Exposure on the Development and Myocardium of Adult Mice: Morphometric Changes, Transcriptional Modulation of Genes Related to Cardiac Dysfunction, and Antioxidant Cardioprotection
Source: Antioxidants (Basel). 2023 Jan 23;12(2):256. doi: 10.3390/antiox12020256 (PMC9952294; doi:10.3390/antiox12020256)
Supplement: Supplementary file 1 [file antioxidants-12-00256-s001.zip › antioxidants-2168086-supplementary.pdf]

**Table S1.** Selected genes for mRNA quantification (Custom TaqMan® Plates). STP – signal transduction pathway

| STP | Gene | Name | NCBI-RefSeq |
|---|---|---|---|
| **Angiogenesis and cell survival** | *Akt1* | V-akt murine thymoma viral oncogene homolog 1 | NM_009652.4 |
| | *Vegfa* | Vascular endothelial growth factor A | NM_001287056.1 |
| **Cell apoptosis** | *Bax* | BCL2-associated X protein | NM_007527.4 |
| | *Fas* | Fas (TNF receptor superfamily member 6) | NM_007987.2 |
| | *Mapk1* | Mitogen-activated protein kinase 1 | NM_011949.3 |
| | *Mapk14* | Mitogen-activated protein kinase 14 | NM_011951.3 |
| | *Trp53* | Transformation related protein 53 | NM_011640.3 |
| **Calcium Kinetics** | *Atp2a2* | ATPase, Ca++ transporting, cardiac muscle, slow twitch 2 | NM_001110140.3 |
| | *Casq2* | Calsequestrin 2 | NM_001355663.1 |
| | *Pln* | Phospholamban | NM_001141927.1 |
| | *RyR-2* | Ryanodine receptor 2, cardiac | NM_023868.2 |
| | *Slc8a1* | Solute carrier family 8 (sodium/calcium exchanger), member 1 | NM_011406.3 |
| **Oxidative stress** | *Cat* | Catalase | NM_009804.2 |
| | *Gpx4* | Glutathione peroxidase 4 | NM_008162.4 |
| | *Hspa1a/b* | Heat shock protein 1A | NM_010479.2 |
| | | Heat shock protein 1B | NM_010478.2 |
| | *Sod1* | Superoxide dismutase 1, soluble | NM_011434.2 |
| **Cardiac hypertrophy** | *Ace* | Angiotensin I converting enzyme (peptidyl-dipeptidase A) 1 | NM_207624.6 |
| | *Ace2* | Angiotensin I converting enzyme (peptidyl-dipeptidase A) 2 | NM_001130513.1 |
| | *Agtr1a* | Angiotensin II receptor, type 1a | NM_177322.3 |
| | *Cabin1* | Calcineurin binding protein 1 | NM_172549.3 |
| | *Chp2* | Calcineurin-like EF hand protein 2 | NM_027363.1 |
| | *Edn1* | Endothelin 1 | NM_010104.4 |
| | *Igf1* | Insulin-like growth factor 1 | NM_010512.5 |
| | *Map3k2* | Mitogen-activated protein kinase kinase kinase 2 | NM_011946.3 |
| | *Myh6* | Myosin, heavy polypeptide 6, cardiac muscle, alpha | NM_001164171.1 |
| | *Myh7* | Myosin, heavy polypeptide 7, cardiac muscle, beta | NM_080728.3 |
| | *Nfatc3* | Nucl. factor of activated T cells, cytoplasmic, calcineurin dependent 3 | NM_010901.3 |
| | *Nppa* | Natriuretic peptide type A | NM_008725.3 |
| | *Nppb* | Natriuretic peptide type B | NM_008726.6 |
| | *Prkca* | Protein kinase C, alpha | NM_011101.3 |
| | *Prkcb* | Protein kinase C, beta | NM_008855.2 |
| | *Prkcg* | Protein kinase C, gamma | NM_011102.4 |
| **Inflammation** | *IL-6* | Interleukin 6 | NM_031168.2 |
| | *Tnfrsf1a* | Tumor necrosis factor receptor superfamily, member 1a | NM_011609.4 |
| | *Tnf* | Tumor necrosis factor | NM_013693.3 |
| **Extracellular Matrix** | *Col1a1* | Collagen, type I, alpha 1 | NM_007742.4 |
| | *Col3a1* | Collagen, type III, alpha 1 | NM_009930.2 |
| | *Mmp9* | Matrix metallopeptidase 9 | NM_013599.5 |
| | *Tgfb1* | Transforming growth factor, beta 1 | NM_011577.2 |
| | *Tnc* | Tenascin C | NM_001369211.1 |
| **Cellular metabolism** | *Gapdh* | Glyceraldehyde-3-phosphate dehydrogenase | NM_001289726.2 |
| | *Hk1* | Hexokinase 1 | NM_001146100.1 |
| | *Ndufa3* | NADH:ubiquinone oxidoreductase subunit A3 | NM_025348.3 |
| | *Pfkm* | Phosphofructokinase, muscle | NM_001163487.1 |
| | *Slc2a1* | Solute carrier family 2 (facilitated glucose transporter), member 1 | NM_011400.3 |
| | *Taz* | Tafazzin, phospholipid-lysophospholipid transacylase | NM_001173547.2 |
| | *Ucp-2* | Uncoupling protein 2 (mitochondrial, proton carrier) | NM_011671.5 |
